# Vertebral Osteomyelitis Secondary to a Retroperitoneal Abscess in an Adolescent Male: A Case Report

**DOI:** 10.7759/cureus.30584

**Published:** 2022-10-22

**Authors:** Fatema H Madan, Mohamed Rahma, Badryia Toorani

**Affiliations:** 1 Orthopaedics and Trauma, Salmaniya Medical Complex, Manama, BHR; 2 Orthopaedics and Trauma, Salmanyia Medical Complex, Manama, BHR

**Keywords:** lumbar spondylodiscitis, spine infections & trauma, spine infections, retroperitoneal abscess, vertebral osteomyelitis

## Abstract

Pyogenic vertebral osteomyelitis (PVO) is included in a spectrum of spine infections called spondylodiscitis. It is commonly caused by hematogenous seeding. The annual incidence of spondylodiscitis is 2.4 per 100,000 person-years and the incidence triples for people over seventy.

We report a case of a previously healthy 14-year-old male with a rare presentation of vertebral osteomyelitis associated with a large retroperitoneal abscess of unknown origin, manifested with flank pain, back pain, and fever, and treated with antibiotics with good short-term outcomes.

We concluded that vertebral osteomyelitis in adolescent patients could manifest with back or flank pain. Moreover, it can occur as an extraperitoneal manifestation of a retroperitoneal abscess.

## Introduction

Although uncommon spinal infections can lead to a serious clinical sequela that often necessitates aggressive medical and surgical treatment, the spectrum of spinal infections includes osteomyelitis, isolated discitis, spondylodiscitis, epidural abscess, and spinal cord abscess. The term spondylodiscitis refers to discitis and vertebral osteomyelitis, as these two conditions can occur in conjunction with each other [1].

Pyogenic vertebral osteomyelitis (PVO) is caused by an infection of the vertebral bodies that can spread to the intervertebral disc spaces [2]. Staphylococcal infections, including *Staphylococcus aureus*, are the most commonly identifiable causative micro-organisms in PVO. It can also be caused by Gram-negative organisms such as *Escherichia coli*, *Pseudomonas aeruginosa*, *Haemophilus influenza*, and *Klebsiella pneumonia* [3].

This paper reports the rare presentation of vertebral osteomyelitis associated with a large retroperitoneal abscess in a previously healthy 14-year-old male. Patient consent for publication was obtained prior to submitting the article.

## Case presentation

A 14-year-old Bahraini male, previously not known to have had any medical illness, had multiple visits to the emergency department with flank pain associated with fever for two months, which increased in severity over the last two weeks prior to the final presentation. Initially, he reported pain in the right flank radiating to the right lower abdomen. He also complained of a loss of appetite but had no history of weight loss, nausea, or vomiting. He reported no history of painful micturition, throat pain, cough, night sweats, joint pain, or skin rashes. His vaccinations were up to date, and he had no recent travel history.

Upon examining the patient, he was fully conscious and oriented. He was sitting in bed with flexed knees while attempting to avoid lying in a supine position as it aggravated the pain. On the first day of the presentation, he was febrile, with a temperature of 38.5 °C and tachycardia, with a heart rate of 140 beats per minute. However, other vitals were within normal limits. Examination of the abdomen revealed a non-distended abdomen, he was voluntarily guarding on palpation, and there was tenderness and rebound tenderness in the right iliac fossa. Moreover, there was tenderness in the right renal angle. Per-rectum examination showed normal sphincter tone.

He had no previous history of any medical illness. Also, there was no family history of diseases or similar symptoms among family members. Also, there was no travel history, previous surgeries, or hospital admissions. He is a middle school student and a football player.

**Figure 1:**
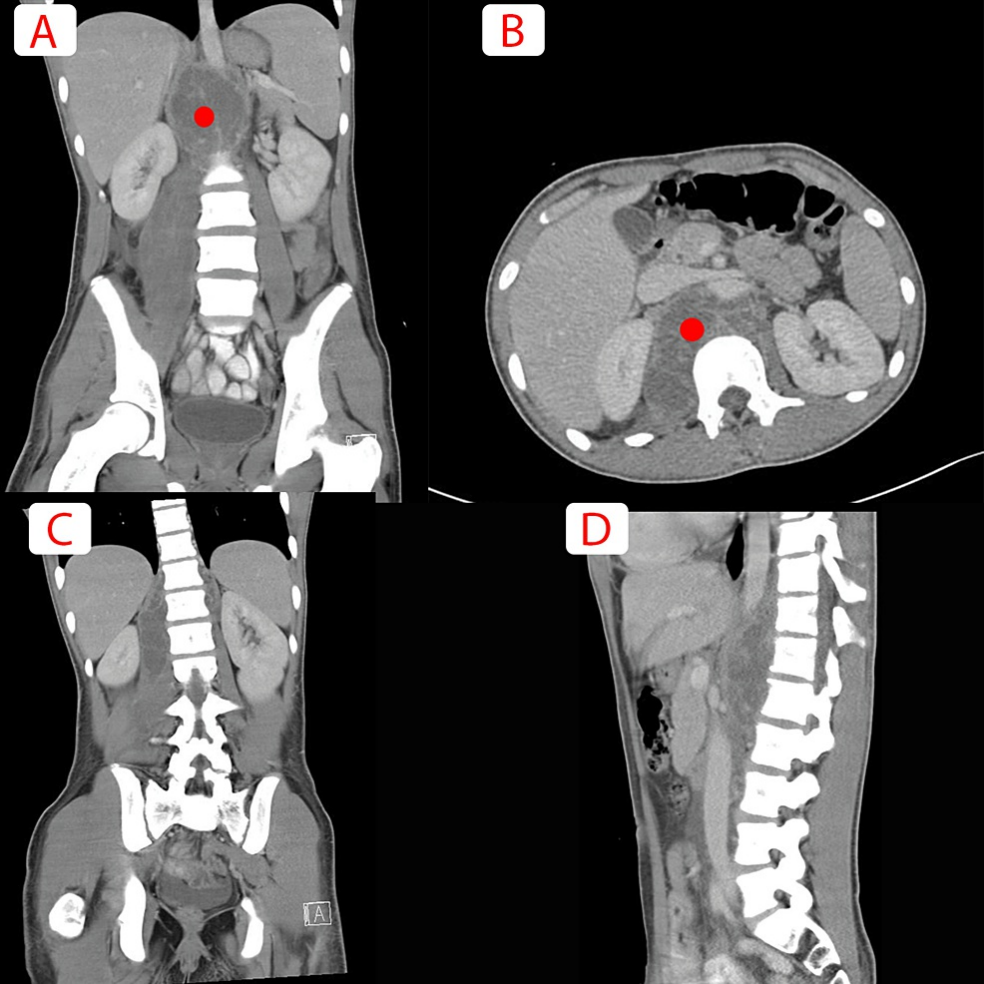
(A) Axial and (B) sagittal view of a CT scan of abdomen an pelvis showing a large thick multiseptated retroperitoneal abscess (red circle localizing the retroperitoneal abscess) extending to the right iliopsoas and iliacus muscle with associated right pelvis and rectovesical pouch free fluid, and a few hypodense, necrotic enlarged retroperitoneal lymphadenopathy. No underlying bony destruction to suggest discitis. C and D are further images showing no vertebral involvement at this stage.

The initial blood panel revealed an elevated white blood cell count of 17.6 10^3^/mm^3^, with neutrophils of 89%, an erythrocyte sedimentation rate (ESR) of 75 mm/hour, and a C-reactive protein (CRP) of 227.1. His peripheral blood culture was methicillin-resistant *Staphylococcus aureus* (MRSA) positive and sensitive to vancomycin, clindamycin, and trimethoprim/sulphamethoxazole. The patient was treated with vancomycin as it had the lowest minimum inhibitory concentration value of 1 µg/ml. A negative acid-fast bacillus assay ruled out tuberculosis from blood and sputum. His urine analysis and renal and liver function tests were within the normal range. Non-contrast computed tomography scan of the abdomen and pelvis showed a large right retroperitoneal abscess 30 cm in size with a small peritoneal extension (Figure 1).

**Figure 2:**
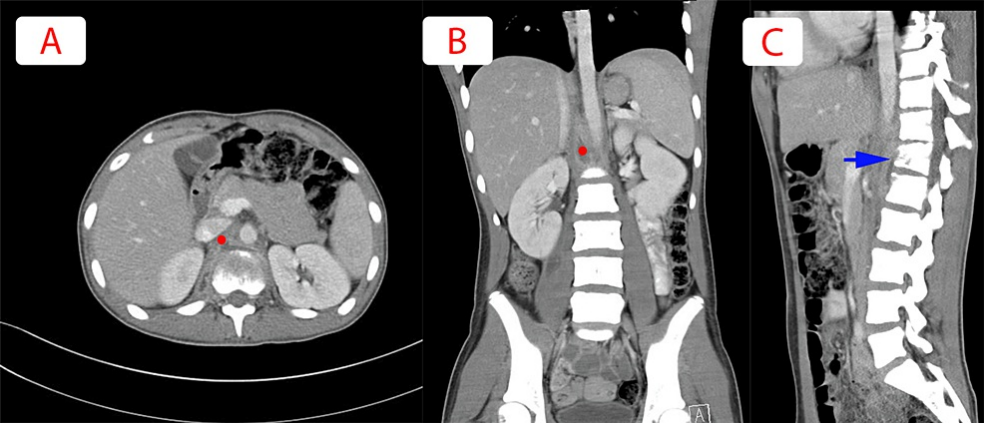
(A) Coronal, (B) axial, and (C) sagittal views of abdomen and pelvis CT scan. The red circle localizing the significant interval resolution of the right psoas and retroperitoneal collection against T12-L1. Blue arrow showing underlying reduction in the height of T12 vertebra with irregularity of endplates and lucencies within the vertebral body and along the anterio-superior aspect of L1 vertebra with slightly reduced interval raising the suspicion for spondylodiscitis.

He was admitted with the impression of a retroperitoneal abscess and was started on intravenous vancomycin. Also, an ultrasound-guided percutaneous drainage procedure was done, and the drained pus culture was positive for *S. aureus* MRSA and was negative for tuberculosis. Thorough laboratory investigations were done throughout his admission. The serological, immunologic, vasculitis, and rheumatoid panels were all negative. The repeated CT scan showed significant interval resolution of the right psoas and retroperitoneal collection against the T12-L1 vertebra (Figure 2). However, a reduction in the size of the T12 vertebra with irregularity of the endplates and lucencies within the vertebral bodies along the anterosuperior aspect of the L1 vertebra was seen. Moreover, the disc spaces were also slightly reduced, suggesting underlying infective spondylodiscitis.

**Figure 3:**
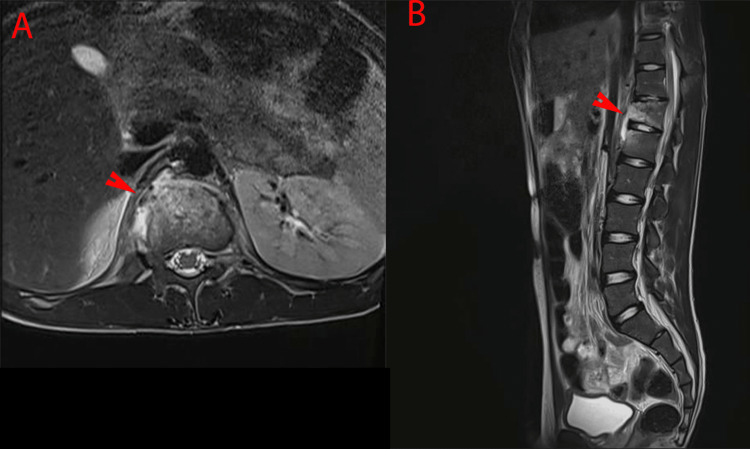
T2 weighted images of thoracolumbar MRI post drainage procedure shows subligamentous extension at the level of T12 and L1 vertebra leading to osteomyelitis of those vertebrae. T12 vertebra showing mild vertebra height reduction with an abnormal signal intensity high in T2, similar findings in the superior anterior end of L1. No definite extension to the neural foramina or the central canal posteriorly. Red arrowheads point to vertebral osteomyelitis.

Thoracolumbar magnetic resonance imaging showed abnormal signal intensity in the T12 and L1 vertebral bodies that are high in T2 and STIR and low in T1 with intense post-contrast enhancement. In addition, we found a right-sided psoas abscess with a subligamentous extension of over 3.7 cm in the longitudinal axis. It was also associated with soft tissue thickening predominantly at the anterior and lateral aspects of the vertebra and disc, with no definite extension to the neural foramina or the central canal posteriorly. The intervertebral disc appeared largely preserved with no definite abnormal signal intensity suggestive of involvement. These findings represent osteomyelitis due to the subligamentous extension of the adjacent psoas abscess (Figure 3).

During his hospital stay, he completed an entire course of intravenous vancomycin, 1 g twice daily. He was also clinically improving, and his inflammatory markers were monitored weekly and showed significant improvement. He was discharged on oral clindamycin. He received an entire course of six weeks of antibiotics. His C-reactive protein was reduced from 227.1 to 17.3 on the day of discharge from the hospital.

**Figure 4:**
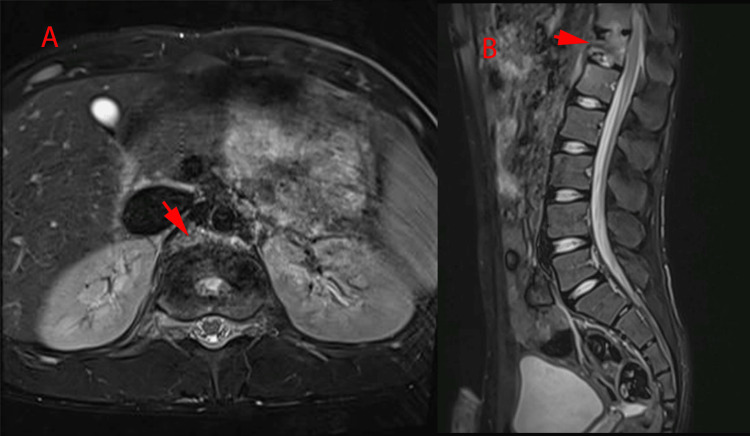
(A) Axial and (B) sagittal view of T2-weighted image of thoracolumbar MR post completion of antibiotics course. Red arrowheads shows interval regression with good response to treatment that shows the process of healing involving the T12 vertebra in the form of sclerotic changes, interval regression in the disc collection involving T11-12 and the anterior sub ligamental collection at the level T12 and L1. Complete resolution of the retroperitoneal collection seen.

On follow-up, a repeat thoracolumbar magnetic resonance imaging after six weeks showed interval regression with a good response to treatment and a healing process seen in sclerotic changes at the level of T12-L1 (Figure 4). The patient described relief from his previous symptoms, and his appetite improved. At five months of follow-up, he had a short-term full recovery and went back to his daily life activities, but was instructed to abstain from athletic activities and to wear a lumbosacral brace for six weeks.

## Discussion

Pyogenic vertebral osteomyelitis incidence is rare as it accounts for 2-5% of all osteomyelitis but can be life-threatening. The annual incidence of acute hematogenous nontuberculous vertebral osteomyelitis is estimated to be around 5 per million patients, with a male predominance. According to a French study, the annual incidence of spondylodiscitis is 2.4 per 100,000 person-years and the incidence triples for people over seventy [4-6].

This condition's presentation is usually delayed due to the mild initial and non-specific clinical manifestations. Delays in diagnosis can result in the spread of the infectious process, resulting in neurological complications [7], and approximately one-third of recovered patients have residual disabilities that severely affect life quality [8].

Bacterial spread occurs through two pathways: either by hematogenous seeding, which is the most common, or direct inoculation through surgical procedures or penetrating trauma, which is rare [2,9]. Presentation of this condition in this age group is rare. Likewise, vertebral osteomyelitis secondary to retroperitoneal abscess is rare, as the most common route of spread is hematogenous seeding. However, it is feasible to know the exact etiology or source of the infection as he had no history of upper respiratory tract infection, urinary tract infection, or skin rashes.

The Infectious Diseases Society of America (IDSA) guidelines for the diagnosis and treatment of vertebral osteomyelitis in adults include antibiotic therapy in the form of parenteral or highly bioavailable oral antimicrobial therapy for six weeks [10]. Many studies compared 12 weeks versus six weeks of antibiotic therapy in patients, and they found that six weeks of antibiotic treatment is not inferior to 12 weeks of antibiotic treatment [11,12]. Surgical intervention is indicated in cases complicated by drainable abscesses, patients with neurological compromise, signs of neural foramina compression, or no improvement with antibiotics [7].

Similarly, cases have been previously reported of vertebral osteomyelitis in similar age groups, such as a 13-year-old girl with vertebral osteomyelitis, similar to our patient, who complained of worsening back pain. However, contrary to our patient, who had negative cultures but was treated with IV antibiotics and improved [7].

## Conclusions

In conclusion, although uncommon, vertebral osteomyelitis can present in adolescent patients manifesting as back pain and fever. The most common route of infection is a hematogenous route, and most commonly it is caused by *S. aureus*. The treatment modality is mainly intravenous antibiotics, but surgical drainage is indicated in cases complicated by drainable abscesses or patients with no improvement with intravenous antibiotics. Moreover, it can occur as an extraperitoneal manifestation of a retroperitoneal abscess.
